# The promise and challenge of spatial omics in dissecting tumour microenvironment and the role of AI

**DOI:** 10.3389/fonc.2023.1172314

**Published:** 2023-05-01

**Authors:** Ren Yuan Lee, Chan Way Ng, Menaka Priyadharsani Rajapakse, Nicholas Ang, Joe Poh Sheng Yeong, Mai Chan Lau

**Affiliations:** ^1^Singapore Thong Chai Medical Institution, Singapore, Singapore; ^2^Yong Loo Lin School of Medicine, National University of Singapore, Singapore, Singapore; ^3^Singapore Immunology Network (SIgN), Agency for Science, Technology and Research (A*STAR), Singapore, Singapore; ^4^Department of Anatomical Pathology, Singapore General Hospital, Singapore, Singapore; ^5^Cancer Science Institute of Singapore, National University of Singapore, Singapore, Singapore; ^6^Bioinformatics Institute (BII), Agency for Science, Technology and Research (A*STAR), Singapore, Singapore

**Keywords:** spatial omics, tumour microenvironment, artificial intelligence, machine learning, deep learning, spatial proteomics, spatial transcriptomics, digital pathology

## Abstract

Growing evidence supports the critical role of tumour microenvironment (TME) in tumour progression, metastases, and treatment response. However, the *in-situ* interplay among various TME components, particularly between immune and tumour cells, are largely unknown, hindering our understanding of how tumour progresses and responds to treatment. While mainstream single-cell omics techniques allow deep, single-cell phenotyping, they lack crucial spatial information for *in-situ* cell-cell interaction analysis. On the other hand, tissue-based approaches such as hematoxylin and eosin and chromogenic immunohistochemistry staining can preserve the spatial information of TME components but are limited by their low-content staining. High-content spatial profiling technologies, termed spatial omics, have greatly advanced in the past decades to overcome these limitations. These technologies continue to emerge to include more molecular features (RNAs and/or proteins) and to enhance spatial resolution, opening new opportunities for discovering novel biological knowledge, biomarkers, and therapeutic targets. These advancements also spur the need for novel computational methods to mine useful TME insights from the increasing data complexity confounded by high molecular features and spatial resolution. In this review, we present state-of-the-art spatial omics technologies, their applications, major strengths, and limitations as well as the role of artificial intelligence (AI) in TME studies.

## Introduction

Tumour microenvironment (TME) plays an important role in disease progression and clinical outcomes. TME is made up of multiple components including fibroblasts, immunosuppressive cells, immune effector cells, and cytokines (1). Specific T-cell subsets, including CD4^+^ helper and CD8^+^ T-cells offer protective immunity (2). On the other hand, tumour-associated macrophages (TAM) which is the most prevalent infiltrating immune cells in the TME can promote tumour growth when accompanied by the activation of fibroblasts. Localization of TAM near invasive borders correlates with unfavorable prognoses in tumors such as colorectal cancer (CRC) (3). Similarly, tumour-associated neutrophils (TANs) can transition from anticancer to pro-tumorigenic phenotypes due to unclear mechanisms (4, 5). TME influences treatment outcomes through multiple mechanisms. In ovarian and lung malignancies, TAM-associated exosomes, which are small membrane-bound vesicles that contain proteins, lipids, and nucleic acids which can be transferred to neighboring cells to influence their physiological behavior, thereby increasing tumour proliferation, apoptosis inhibition and drug resistance (6, 7). TANs were found to have tumour-promoting effects in the lung TME, leading to unfavorable immunotherapy (IO) outcomes (8). Higher mast cell levels in TME were associated with higher PD-L1 expression (9) indicating potential impact on immune checkpoint inhibitor (ICI) efficacy. Increased CD4^+^ helper T-cells have been postulated to improve IO outcomes through enhancing cytotoxic T-cell response (10). A previous study has shown that phenotypically defined T-cell subsets, rather than overall T-cells, may be useful in predicting therapy outcomes (11).

**Figure 1:**
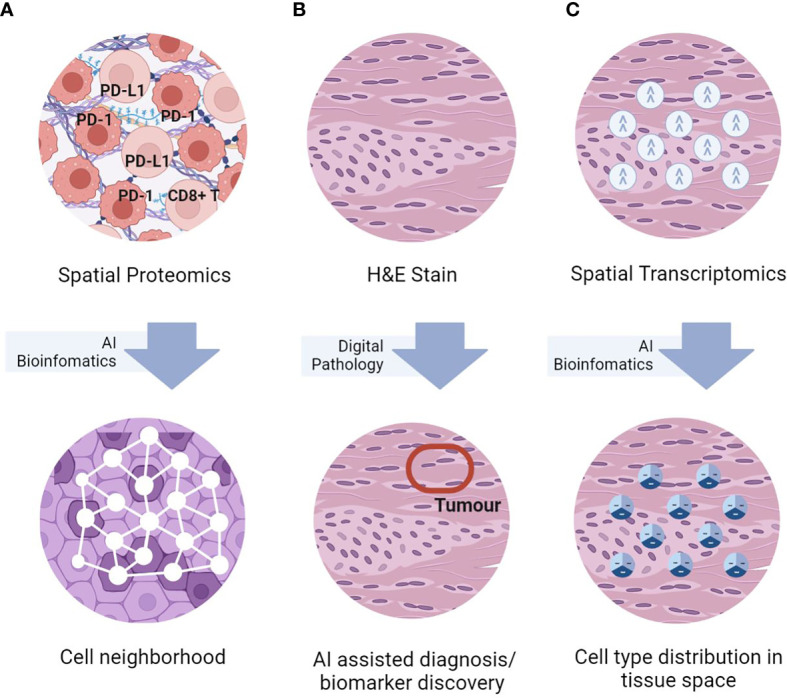
Simplified graphical representations of the three key spatial omics technologies, namely **(A)** spatial proteomics assays for *in-situ* single-cell phenotyping using surface marker; **(B)** H&E staining for histomorphological assessment, and **(C)** spatial transcriptomics for *in-situ* transcriptomics characterization, and representative TME analyses enabled by AI bioinformatics.

While traditional immunohistochemistry (IHC) and hematoxylin and eosin (H&E) tissue staining have been used routinely for tumour diagnosis, their low-content limits usefulness in TME analysis. On the other hand, high-throughput technologies such as single-cell RNA-sequencing (scRNAseq) and flow cytometry, despite allowing for high-content molecular profiling, they lose spatial information during tissue dissociation. Additionally, experimental tissue dissociation may result in unexpected cell phenotypic alterations unrepresentative of the actual TME. To address these issues, novel tissue-based spatial omics approaches have recently been developed (12). These advanced spatial techniques enable deep phenotyping, such as distinguishing M1- from M2-polarized macrophages (13) and mature from immature myeloid cells (14), which cannot be achieved with IHC and H&E alone. Additionally, by conserving the spatial information, these techniques allows identification of unique spatial patterns of immune cells in TME with novel biological significance, such as TAM-associated cellular neighborhoods with different antitumor characteristics (15), TMEs with various TAN subtypes linked to prognosis and survival (16), differing states of T cell dysfunction contributing to tumour propagation (17), and ligand-receptor cell interactions (18) associated with various prognoses and treatment outcomes (19). In this review, we will introduce and discuss how state-of-the-art spatial proteomics (SP), spatial transcriptomics (ST) and the utilization of artificial intelligence (AI) approaches that can benefit TME analysis (**Figure 1**). We will also provide our perspectives on the challenges and future development needed to advance the field of spatial omics.

## SP techniques

In this section, we discuss the two major groups of SP techniques, namely fluorescent-labelling and metal isotope-labelling assays (**Table 1**), which differ in the number of plexing, throughput, resolution, and cost (39).

**Table 1:** Summary of SP and ST techniques as well as their associated applications in TME analysis.

Technique	Detection	Vendor	Profiling technology	Plexing	Tissue Type	Companion analytical software	Key TME applications
Opal-based multiplex IHC	Proteins	Akoya Biosciences	Fluorescence-labelling reagent kits	9	FFPE	InForm	Deep phenotyping of macrophage polarization state, myeloids, T-cell subsets (15) in CRC (14) and pancreatic cancers (20).
COMET	Proteins	Lunaphore	Cyclic fluorescence labelling platform	40	FF/ FFPE	Phenoplex	Identification of spatially restricted myeloid and T regulatory cells in primary lung cancers (21)
PhenoCylcer	Proteins	Akoya Biosciences	Cyclic fluorescence labelling platform	50	FF/ FFPE	PhenoCycler MAV software	Identification of distinct cellular neighbourhoods with survival association in CRC (22)
IMC	Proteins	Standard BioTools	Metal-based labelling	50	FF/ FFPE	Phenoplex	Study of heterotypic neighbourhoods of a specific myofibroblast phenotype in breast cancer (23)
MIBI	Proteins	Ionpath	Metal isotope-labelling	100	FF/ FFPE	MIBItracker Software	Study of the spatial organization and immuno profile of 15 tumor types, revealing infiltration of CD8^+^ cytotoxic T cells and CD68^+^ macrophages in ovarian serous carcinoma TME (24); spatial enrichment analysis revealed that tumors were immune mixed and compartmentalized with varying expression of PD1, PD-L1, and IDO on a cell-type and location-specific basis, where highly ordered structures with PD-L1 and IDO along the tumor-immune border served as a hallmark of tumor compartmentalization in a triple-negative breast cancer patients (25).
Visium	RNA	10X Genomics	NGS	18000	FF/ FFPE	Spaceranger, Loupe browser	Identification of enrichment of B-cell maturation and anti-tumorigenic antibody production within TLS+ compartment and positive association with clinical outcomes of renal cell carcinoma (26); study of long-term effects of SARS-CoV-2 in hepatocellular carcinoma and CRC, revealing persistent B-cell immune responses and improved in-silico IO-response scores in SARS-CoV-2-rich tissue regions (27).
GeoMx DSP	Proteins/ RNA	nanoString	NGS	100+ (proteins)/ 18000+ (RNA)	FF/ FFPE	GeoMx DSP online suite, GeoMx tools (R package)	Study of the association between CD66b expression within the CD45+CD68 compartment and ICI resistance, which however, not observed in ICI-untreated lung cancer patients (28); characterization of 4 molecularly unique compartments: tumor, leukocyte, macrophage, and immune stroma where different biomarkers in specific compartments show improved survivals in heck and neck SCC (29)
Slide-Seq/ Seeker	RNA	Curio Bioscience	NGS	whole-transcriptome	FF	–	Identification of cellular neighbourhood archetypes associated with tumour progression and malignancy (30); spatial TCR clonotypic effect of IO treatment in metastatic lung cancer (31)
Stereo-seq	RNA	Beijing Genomic Institute	NGS	whole transcriptome	FF/ FFPE	Stereo-seq Analysis Workflow (SAW) software suite	Study of hepatocellular carcinoma shows that elevated expressions of Serum Amyloid A observed in hepatocytes located near invasive fronts of the tumor were linked to increased macrophage recruitment, and were associated with a negative prognosis in intrahepatic cholangiocarcinoma (32); study of CRC tissue identified locoregional “warmed-up” immune response in predefined “cold” tumor where the "warmed-up" signature genes were found to be indicative of improved overall survival in patients with CRC (33).
MERFISH/MERSCOPE	RNA	Vizgen	Imaging	~10,000	FF/ FFPE	–	Identification of a shift in immune spatial organization between tumour subtypes, namely human mismatch repair deficient and proficient tumours (34)
CosMx	Proteins/ RNA	nanoString	Imaging	100/ 1000+	FF/ FFPE	AtoMx Spatial Informatics Platform	Quantification of proteins in NSCLC and BC tissues down to subcellular resolution for the identification of different cell types, unique TMEs, and ligand-receptor pairs (35); Study of relationships between high-dimensional cellular heterogeneity and spatial organization of cells within renal cell carcinoma tissues (36)
Xenium	RNA	10X Genomics	Imaging	280 and 100 more customizable targets; the non-destructive nature allows post-Xenium H&E and IF staining on the same section rendering additional SP and histological information	FF/ FFPE	Xenium Explorer	Identification of novel markers at subcellular level responsible for the transition between ductal carcinoma in situ (DCIS) and invasive cancer of human breast tissues where the myoepthelial layer is broken (37); identification and interrogation of the cellular composition and differentially expressed genes among the 3 molecular subtypes of BC (low, high-grade DCIS, and invasive cancer) through integrating Xenium with H&E and IF data (38).

### Fluorescence-labelling techniques

OPAL-based multiplex IHC (mIHC)/immunofluorescence (IF) technique allows staining up to 9 markers on a single formalin-fixed, paraffin-embedded (FFPE) tissue section through tyramide signal amplification (TSA). It represents one of the most popular SP techniques for (i) its widely validated consistency against conventional IHC (40, 41); (ii) autostainer availability, particularly BondMax (Leica Biosystems, Germany) for staining consistency (39, 42–45); and (iii) clinical feasibility and usefulness (46–48). The technique has been widely applied for investigating the complex TME through enabling accurate and deep cell phenotyping (e.g., macrophage polarization states, myeloid cell maturity and immunosuppressivity, and T cell phenotypes) (13), revealing the spatial heterogeneity of immune cells (49–51), and characterizing immune localization patterns associated with patient survivals or treatment outcomes (20). Using proximity analysis, Feng et al. showed that hampered survival outcomes of oral squamous cell cancers (SCC) was associated with CD8^+^ T-cells surrounded by immunosuppressive FoxP3^+^ or PD-L1^+^ cells (52); Väyrynen et al. showed that CRC patients with mature monocytic cell (CD14^+^HLADR^+^) closer to tumour cells harbored better survival (14). One disadvantage of OPAL-based mIHC/IF technique is the possibility of physical steric hindrance caused by multiple antibodies at a single site, resulting in noisy signals (53).

To enable comprehensive immune profiling, hyper-plex cyclic mIHC/IF techniques have been developed, including COMET (Lunaphore, Switzerland) and PhenoCycler (Akoya Biosciences, USA). COMET provides an automated workflow cycling through staining, imaging, and elution of 3 markers each time, up to 40 markers in the same tissue section, whereby fluorophores are directly attached to secondary antibodies without TSA. This approach not only reduces steric hindrance with lesser markers per cycle, but also enhances signal stability through reducing incubation time (i.e., tissue exposure time to harsh reagents). Using a 40-plex COMET assay, Almeida et al. found that myeloid and T regulatory cells were spatially restricted in primary lung cancers (21). Using machine learning (ML), the authors also identified distinct subsets of myeloid cells within the same TME.

To further reduce the steric hindrance effect, PhenoCylcer (formerly CODEX) replaces the large molecular secondary antibodies in the OPAL approach with DNA-conjugated antibodies tagged to fluorescent reporters, allowing staining of up to 50 markers (3 per cycle). Applying tensor decomposition, cell-type differential enrichment and canonical correlation analysis on PhenoCycler data, Schürch et al. found 9 distinct cellular neighborhoods associated with survival outcomes which were conserved across 35 CRC samples (22).

### Metal isotope-labelling techniques

Metal-based methods employ stable metal isotopes to replace the fluorophores (54), where protein expression is measured by detecting isotope signals using laser scanning or ion beams. Autofluorescence and background noise can be considerably reduced with endogenous metals (55). Imaging mass cytometry (IMC) (Standard BioTools, USA) uses high-resolution scanning laser ablation (a fixed lateral resolution of 1,000nm) followed by mass cytometry to quantify up to 50 markers at subcellular resolution using fresh frozen (FF)/FFPE tissues (56–58). Using 37-plex IMC, Ali et al. identified heterotypic neighborhoods of a specific myofibroblast phenotype which was associated with poor outcomes in breast cancer (BC) (23).

Another metal-based method, multiplexed ion beam imaging (MIBI) (Ionpath, USA) uses a tuneable ion beam voltage and mass spectrometry to detect molecules of interest (24), analyzing up to 100 markers at subcellular resolution using FF/FFPE tissues (25, 59, 60). Ptacek et al. validated the robustness, sensitivity, and reproducibility of MIBI against individual IHC stains (24), while Angelo et al. and Rost et al. tested the consistency of MIBI against IHC procedures for estrogen receptor alpha, progesterone receptor and human epidermal growth factor receptor 2 using FFPE BC samples (60, 61). ML techniques have been extensively used in these MIBI studies for rapid exploration and analysis of data for novel discoveries. For examples, Keren et al. developed a computational pipeline to chart the immune landscape in triple-negative breast cancer. In their pipeline, they employed multiple ML techniques such as DeepCell for cell segmentation, *k*-nearest neighbor algorithm for noise filtering, quantile normalization for batch effects correction, and hierarchical clustering to identify unique and shared spatial interactions among patients (25). Padmanabhan et al. built multiple DL models for segmentation of cells and regions, and for cell classification. A containerized cluster platform that can run a workflow comprising of pre-trained DL models as directed acyclic graph has been used to accelerate the discovery of associations and spatial patters in TME (26). Authors in (23, 60) used CellProfiler, an image analysis tool encompassing number of ML algorithms such as random forest, principal component analysis, and neural networks (NN) to understand phenotypic impact of genomic alterations and to gain new insights from the combination of tissue microarchitecture with multiplexed protein expression patterns, respectively.

## ST techniques

ST methods can be broadly categorized into next-generation sequencing (NGS)-based and imaging-based methods, measuring either near whole-transcriptome at multi-cell (10s to hundreds) resolution or selected genes at subcellular spatial resolution. NGS-based methods acquire spatial transcriptomic data by attaching and sequencing unique barcodes to cell subsets in designated tissue areas such as a lattice of evenly spaced spots, user-marked regions, or marker-stained regions. This untargeted nature of NGS based methods make them suitable for exploratory studies (62). Imaging-based methods quantify transcripts *in-situ* through direct imaging of fluorescence dyes of the nucleic acid bases (termed *in-situ* sequencing) or the target-specific/bound fluorophore (termed *in-situ* hybridization (ISH)).

### NGS-based ST techniques

Visium Spatial Gene Expression (10x Genomics, USA) enables genome-wide ST profiling of FF/FFPE tissues. The slide capture area (6.5mm^2^) contains ~5,000 spots, each with a 55 μm diameter. Using Visium, Meylan et al. examined the B-cell response within intratumoral tertiary lymphoid structures (TLS) in renal cell carcinoma, and found positive clinical outcomes associated with intratumoral TLS+ regions enriched with B-cell maturation and anti-tumorigenic antibody production (26); Lau et al. examined the long-term effects of SARS-CoV-2 in hepatocellular carcinoma and CRC, revealing persistent B-cell immune responses and improved in-silico IO-response scores in SARS-CoV-2-rich tissue regions (27). Another genome-wide ST method, Slide-seq (Applied Biotechnology Laboratory, UK), offers higher resolution (10 µm) read-outs with a comparable capture area (in mm-range), but is limited to FF tissues (30, 63, 64). Using Slide-seq, Avraham-Davidi et al. revealed three distinct cellular neighborhood archetypes associated with tumour progression and malignancy (30). Liu et al. further developed Slide-T cell receptor (TCR)-seq and identified 1,132 unique clonotypes, some localized in restricted tissue compartments in metastatic lung cancer post anti-PD-1 therapy, revealing spatial clonotypic effect of IO treatment (31).

GeoMx digital spatial profiler (DSP) (Nanostring, USA) is capable of simultaneous ST (thousands to tens of thousands of genes) and SP (1 nuclear and 3 surface markers) profiling of FF/FFPE tissues of up to 36.2mm x 14.6mm in size (65). RNAs and proteins are quantified through oligonucleotide tagging (with RNA probes or antibodies, respectively), photocleaving, and sequencing. DSP studies showed that higher lymphoid infiltrates and T-cell clonality in the TME were associated with improved IC efficacy (66, 67); CD66b expression in the CD45^+^CD68 molecular compartment was linked to IO therapy resistance in lung cancers (28); B2M and CD25 levels in tumour and CD11c in stroma were correlated with prolonged survival in head and neck SCC (29).

To address key shortfalls of the abovementioned ST methods (i.e., the lack of single-cell resolution read-outs), Stereo-seq (BGI, China) offers unbiased whole-transcriptomic profiling at subcellular resolution with a maximum 1 cm^2^ capture area on FF/FFPE tissues. Using Stereo-seq, Wu et al. showed that poorer prognoses of intrahepatic cholangiocarcinoma and hepatocellular carcinoma were associated with tumour boundaries enriched with damaged hepatocytes, or serum amyloid A overexpression in invasive fronts (32); Zhang et al. found a locoregional immune “warmed-up” phenotype with enhanced cytokine secretion and upregulated MHC-II expression in a predefined “cold” tumour of colorectal adenocarcinoma (33). A major challenge with Stereo-seq analysis lies with assigning pixel-level signals to individual cells (32).

### Imaging-based techniques

Multiplex error robust fluorescence *in situ* hybridization (MERFISH) (Vizgen, UK) uses a combinatorial barcoding approach and sequential rounds of imaging to decode the barcode and its associated gene expression. The barcoding system confers error robustness through assigning the erroneous readout to the nearest correct barcode. MERFISH allows profiling of up to tens of thousands of RNA species at subcellular resolution, with a maximum 1 cm^2^ capture area of FF/FFPE tissues. Using a 450-gene MERFISH panel, Price et al. reported a shift in immune spatial organization between the two tumour subtypes i.e., human mismatch repair deficient and proficient tumors, opening new avenues for tumour subtype-specific treatment strategies (34).

Two other multi-spatial omics methods, namely CosMx spatial molecular imaging (SMI) technique and Xenium (higher-resolution advancement from DSP and Visium, respectively), enable simultaneous ST and SP profiling of FF/FFPE tissues at subcellular resolution. CosMx SMI allows up to 1000-plex and offers 64 validated protein analytes (35, 36). Using CosMx, He et al. (35) evaluated 980 RNAs and 108 proteins in lung cancer and BC tissues, identifying over 18 different cell-types, 10 unique TMEs, and 100 ligand-receptor pairs. While Xenium offers a 280-plex human breast panel and 248-plex mouse brain panel, with additional 100 customizable targets. Due to its non-destructive nature, Xenium allows post-ST H&E staining and IF on the same section, offering additional SP and histological data. Using Xenium, Henley et at. revealed that invasive fronts of ductal carcinoma *in situ* (DCIS) BC were characterized by disrupted myoepithelial layers, and low KRT14 expression which were also positive for progesterone receptor (37); Janesick et al. predicted the hormone receptor status of three BC subtypes (low-grade and high-grade DCIS, and invasive carcinoma) whose molecular signatures were also characterized using whole-transcriptomics Visium on adjacent tissue sections (38).

## AI-enabled TME analysis

### Digital pathology

With recent advancements in imaging techniques and computer vision, DP has greatly emerged as a useful diagnosis assisting and prediction tool (68), alleviating the high labor cost and interobserver variability issues faced by conventional microscope-based approach (69–73). While H&E-stained histomorphology images remain the main imaging modality in DP, the use of mIHC/IF to enable subcellular molecular profiling has become popular (74).

Numerous studies use deep learning (DL) models to augment DP, greatly advancing TME analysis. DL-based cell segmentation algorithms, such as Cellpose (75) and Stardist (76), enable identification of individual nuclei, facilitating downstream cell phenotyping. Supervised DL algorithms have been developed to differentiate benign from malignant cells, and immune from stromal cells (71, 77–79). These approaches are limited by the availability of training labels, resulting in the development of unsupervised approaches (80). Novel AI approaches, such as Ronteix (81), for investigating cell-to-cell interaction have also attracted increasing attention. Besides, as image quality and stain consistency impact the performance of DP studies, several AI algorithms have been developed for stain normalization through color deconvolution (82), clustering in the hue-saturation-value color space for color separation (72) or DL-empowered stain-to-stain translation (83).

### SP analytic methods

SP analysis involves image pre-processing to remove background or technical noise, cell segmentation, feature extraction (such as signal intensity, cellular area, and shape), cell phenotyping and spatial analysis (**Supplementary Table 1**). While image pre-processing steps differ across fluorescence-based and metal-based assays, downstream spatial analysis using extracted cell-level data are largely similar.

Composite multi-spectral images generated with fluorescence-based techniques are firstly unmixed whereby the pixel values are decomposed into the constituent pure spectrum (i.e., protein markers). Spectral unmixing can be done using software like inForm (Akoya Biosciences). Similarly, background subtraction and noise removal are critical steps in pre-processing the multi-channel images acquired from metal-based techniques; each channel portraying the abundance of a protein. Moreover, technique-specific filtering may also be needed – specifically, aggregate removal in MIBI data to eliminate unwanted effects from antibody aggregation, and hot pixels filtering to remove IMC-specific noise (25, 84)

Various cell (or object) segmentation algorithms are deployed in different image analysis tools. To this end, CellProfiler (85) offers several classical image processing approaches; whilst Ilastik (86) offers pixel-based random forest and NN approaches accounting for texture and context that can better identify cells, where both methods require user-input labels such as nuclei and background. Segmentation masks generated by Illastik can serve as the training labels in CellProfiler. These cell segmentation algorithms have been integrated into end-to-end SP analysis pipelines, including IMC Segmentation (87) and its dockerized counterpart, Steinbock (84), adding on to the built-in DL-based Mesmer method (88). More generic image analysis tools including QuPath (89) and ImageJ (90) provides built-in cell segmentation algorithm and allows customized algorithms such as Stardist (91, 92).

There are currently two main cell phenotyping approaches, namely user-input thresholding or rule-based approach, and ML-based supervised approach which require cell label training (93). Using Halo (Indica Labs), Ozbek et al. (94) built a T-cell classifier and computed the densities of 8 different T-cell phenotypes in the tumour epithelial and stromal regions in prostate cancer. Furthermore, dedicated tools for proximity analysis, such as SPIAT (95), HistoCAT (56), imcRtools (84) and Cytomapper (96), have also been developed. These tools enable inter-cellular distance computation, touching-cell counts, cell neighborhood identification, cell-type mixing score, spatial point pattern measures (such as K-cross function), spatial heterogeneity (such as entropy), and immune gradients across tumour margins. Like many methodology studies, these works largely focused on demonstrating evident spatial immunological scenarios in individual cases. For instance, in the SPIAT work, it showed that tumour cells were closely interacting with CD3^+^CD4^+^ and CD3^+^CD8^+^ cells in one prostate cancer sample, while showing the high levels of SOX10^+^ tumour cells did not co-exist with the CD4^+^ immune cells in another prostate cancer sample. Nonetheless, HistoCAT study demonstrated a real oncology case wherein it revealed the enrichment and depletion of cell-cell interactions was associated with breast cancer development.

### ST analytic methods

Several open-source R tools, such as Seurat (97), standR (98), GeoMxTools (99) and Giotto (100), enable end-to-end ST analysis, from data preprocessing (read mapping and quality checking), spatial clustering, spatially variable gene (SVG) identification, cell-type deconvolution to cell-cell communication. Besides these tools, various algorithms to enhance the performance of individual steps have emerged (**Supplementary Table 2**).

Spatial clustering groups spots (neighboring cells) with similar transcriptional profile and characterizes unique transcriptomics niche of the TME (101). These include autoencoder-based methods [such as STAGATE (102), SEDR (103), MAPLE (104), and conST (105)], deep convolution neural network (CNN) methods [such as coSTA (106), RESEPT (107), spaGCN (108), stLearn (109) and spaCell (110), and probabilistic methods (BayesSpace (111) and PRECAST (112)]. SVGs are genes with expression patterns significantly dependent on their spatial locations in the tissues. These include a neural network (NN) method called SOMDE (113); regression modelling methods such as SPARK (114) that uses a generalized linear regression to model the mean-variance relation of NGS-based or imaging-based ST data; SpatialDE (115) uses Gaussian process regression model to decompose gene expression variability into spatial and non-spatial components, tested on SeqFISH and MERFISH data; scGCO (116) addresses the key challenge in SVG analysis, i.e., scalability, by employing a hidden markov random field-based probabilistic graph model, tested on SeqFISH, MERFISH and, 3D ST data (STARmap) (117).

Cell-type deconvolution infers cell composition of the multi-cell ST data, facilitating cell-type specific analysis. These include a Bayesian modelling method called DestVI (118); methods that infer spatial cell composition from scRNAseq data such as CellDART (119) and Tangram (120); a graph-based CNN method called DSTG (121) which was used to uncover cell states of pancreatic tumor tissues. On the other hand, the ability of ST to localize gene expression to specific cell phenotypes in the TME allows effective characterization of cellular communication, which is either through cell-cell direct contact or cell signaling of neighboring cells (122). Analytic tools developed for cellular communication include a scalable random forest-based method called MISTy (123), tested on human BC Visium data; a graph NN method called NCEM (124), tested on MERFISH, PhenoCylcer, and MIBI; a graph CNN model based on a curated list of interacting ligands and receptors, called GCNG (125), tested on SeqFISH and MERFISH.

Aforementioned methods have been mainly focused on showcasing specific ST analytic methods. Studies that involve real oncology use cases are given in (103, 111, 112). In the work of SEDR, the authors analyzed the role of immune microenvironments on tumor invasiveness by clustering the TME into pro-inflammatory and anti-inflammatory regions (103); the authors of PRECAST revealed distinguished tumor/normal epithelial regions in hepatocellular cancers that associated with different signaling pathways, providing higher resolution analysis of the dynamics of tumorigenesis (112); using BayesSpace, Zhao et al. found that a higher level of chemokine activity at the tumor border and an elevated level of metastatic activities at the tumor center that could aid in clinical analysis of cancer metastasis (111).

## Discussion and future perspectives

Significant advancements in spatial omics and computational techniques have unraveled many previously underappreciated roles of immune contexture in cancer progression, immune evasion, and treatment effect, enhancing our understanding of cancer immunology and helping to pave the way towards precision medicine through developing novel therapeutic targets and spatial biomarkers. Increasing evidence show that the phenotypic and functional states of cells, and thus their anti-tumorigenicity, are determined collectively by the DNA, RNA, and protein expression (126–129). Nonetheless, alternative computational solutions for integrating multiple single-spatial omics data represent a valuable resource given tremendous data have been generated separately and available in the public domain. It is also worth to note that recent development of computational methods for cohort analysis reveals important clinical implications by associating immune spatial patterns with treatment response (130, 131).

In our perspective, several challenges in spatial omics need to be addressed. Firstly, advancement in antibody development, automated workflow, image scanning quality and speed, and multi-omics integrative algorithms are needed to enhance robustness, dimensionality, and spatial resolution. Secondly, consistent and quality data is a prerequisite for clinical translation. Several taskforces, such as the Society for Immunotherapy of Cancer (132) and the Joint Effort to Develop Multiplex Immunofluorescence Standards (133), gather international efforts to standardize the workflow of OPAL-based assays, with similar efforts needed for other spatial omics techniques. Thirdly, existing computational tools often require extensive user inputs, such as number of clusters or neighbors, and distance threshold, which hinders adoption. Finally, effective cross-spatial-modality data integration and results interpretation for comprehensive understanding of the biological system remains challenging, largely due to the variations in image format, scanning techniques, sample handling as well as the demanding requirement of computing power and data storage. When these challenges are addressed, robust, affordable, and insightful spatial TME studies may then be possible in helping advancing precision cancer immunology.

## Author contributions

RL and CN contributed equally. All authors contributed to the article and approved the submitted version.
