# Supplementary material for: The promise and challenge of spatial omics in dissecting tumour microenvironment and the role of AI
Source: Front Oncol. 2023 May 1;13:1172314. doi: 10.3389/fonc.2023.1172314 (PMC10183599; doi:10.3389/fonc.2023.1172314)
Supplement: Supplementary file 1 [file Table_1.docx]

Supplementary table 1. Summary of spatial proteomics analysis tools

| **Software names** | **Description** | **Image analysis tasks** | **Interface/programming language** | **Code availability** |
| --- | --- | --- | --- | --- |
| ImageJ | A user-friendly, open-source image processing and analysis software for analysing biomedical images | Background subtraction, noise removal, image (cell/ object) segmentation, morphological and texture analysis, object tracking, quantification, and statistical analysis | Graphical user interface (GUI) with mainly Java codebase; scripting using ImageJ Macro Language | https://github.com/imagej/imagej2 |
| QuPath | A user-friendly, open-source digital pathology software for analysing and interpreting large sets of whole slide images efficiently | Background subtraction, noise removal, nucleus detection, cell segmentation, machine learning-based cell/tissue (object) classification, annotation and classification of histopathological images | GUI with mainly Java codebase; scripting using Groovy | https://github.com/qupath |
| inForm | A user-friendly platform for extracting quantitative information on biomarkers and cellular features of multiplexed biomedical imaging data | Background subtraction, noise removal, spectral unmixing, cell segmentation, spatial statistics and cell phenotyping | GUI | Not available; commercial software by Akoya Biosciences |
| Halo | A digital pathology platform with advanced image analysis tools for more accurate and efficient pathology diagnosis and research using AI | Background subtraction, noise removal, automated cell counting, cell segmentation, cell profiling, cell proximity computation, tumour identification and batch analysis | GUI | Not available; commercial software by Indica Labs |
| CellProfiler | A widely used open-sourced platform for automation of image analysis tasks and execution of complex image analysis protocols | Background subtraction, noise removal, cell segmentation, object detection, and measurement of phenotypic features | GUI or command-line interface (CLI) with mainly Python codebase; Python package; ImageJ macro | https://github.com/CellProfiler/CellProfiler |
| Ilastik | A high throughput, open-source software for enabling non-expert to perform image analysis and machine learning | Background subtraction, noise removal, and pixel-based cell segmentation using machine learning approaches (Random Forest, Neural Network); segmentation masks can be used for cell segmentation in CellProfiler | GUI with mainly Python codebase | https://github.com/ilastik/ilastik |
| IMCSegmentation | A multiplexed image segmentation pipeline based on CellProfiler for segmentation and Ilastik for machine learning to segment the image into different components, such as cells or subcellular structures | A streamlined tool for imaging mass cytometry (IMC) analysis, incorporating background and noise removal, cell detection (using built-in DL-based algorithm, CellProfiler or Ilastik), object detection and feature measurement | Python and Jupyter Notebook | https://github.com/BodenmillerGroup/ImcSegmentationPipeline |
| Steinbock | A dockerised version of IMCSegmentation for IMC visualisation and data analysis | Image preprocessing, pixel classification, image segmentation, object measurements, data export, and visualisation of multiplexed tissue image | Python package with CLI; Docker container with supported third-party software | https://github.com/BodenmillerGroup/steinbock |
| HistoCAT | A software for interactive analysis of multiplexed bioimaging data using advanced clustering algorithms to separate and identify different cell populations | Data visualisation, downstream single-cell analysis of IMC data including intercellular distance computation, touching-cell counts, cell neighbourhood identification, cell-type mixing scores, spatial point pattern measures, spatial heterogeneity, and immune gradient across tumour margins | GUI including web platform and desktop application | https://github.com/BodenmillerGroup/histocat-web |
| imcRtools | A comprehensive toolkit for handling and analysing large scale IMC dataset using various statistical and computational methods to generate meaningful biological insights | Data pre-processing, quality control, visualisation, and spatial mapping, clustering and differential analysis of IMC data | R package | https://github.com/BodenmillerGroup/imcRtools |
| Cytomapper | A toolkit for visualising highly multiplexed, spatially resolved imaging data that can be visualised across multiple length-scales | Visualisation of pixel-level information across multiple channels, display of cell-level information on segmentation masks, gating and visualisation of single cells | R package | https://github.com/BodenmillerGroup/cytomapper |
| SPIAT | A computational method for analysing high dimensional, spatially resolved data from tissues to reveal the spatial organisation of the tissues and the interplay between different cell types | Downstream single-cell analysis of data generated from single-cell spatial proteomics platforms (e.g., OPAL, CODEX, MIBI); includes six analysis modules that allow visualization, calculation of cell colocalization, categorization of the immune microenvironment relative to tumour areas, analysis of cellular neighbourhoods, and the quantification of spatial heterogeneity | R package | https://github.com/TrigosTeam/SPIAT |

Supplementary table 2. Summary of spatial transcriptomics analysis tools

| **Key analysis tasks** | **Deep Learning (DL) components** | **Spatial Transcriptomic (ST) analytic algorithm** | **Description** | **Programming language** | **Codes availability** |
| --- | --- | --- | --- | --- | --- |
| End-to-end ST analysis | Not Applicable | Seurat | For analysing Visium data | R | <https://github.com/satijalab/seurat> |
|  |  | standR | For analysing GeoMx DSP data | R | <https://github.com/DavisLaboratory/standR> |
|  |  | GeoMxTools | For analysing GeoMx DSP data | R | <https://github.com/Nanostring-Biostats/GeomxTools> |
|  |  | Giotto | For integrative analysis and visualisation of different types of ST data | Mainly R | <https://github.com/RubD/Giotto> |
| Clustering | Autoencoder | MAPLE | A hybrid DL and Bayesian modelling framework for multi-sample analysis | R | <https://github.com/carter-allen/maple> |
|  |  | STAGATE | Adaptive graph attention autoencoder for spatial domain identification | Python | <https://github.com/zhanglabtools/STAGATE> |
|  |  | SEDR | A deep autoencoder network for learning a gene representation, and a variational graph autoencoder network for embedding the spatial information | Python | <https://github.com/JinmiaoChenLab/SEDR> |
|  |  | conST | An interpretable multi-model contrastive learning framework for gaining insights on tumour microenvironment and cell-cell interaction | Python | <https://github.com/ys-zong/conST> |
|  | Convolutional Neural Network (CNN) | CoSTA | An unsupervised approach to learn spatial similarities between gene expression profiles via CNN clustering | Mainly Python | <https://github.com/rpmccordlab/CoSTA> |
|  |  | stLearn | For cell type identification, spatial trajectory reconstruction, and the study of cell-cell interactions within a dissociated tissue sample | Mainly Python | <https://github.com/BiomedicalMachineLearning/stLearn> |
|  |  | RESEPT | For characterising and visualising tissue architecture of ST | Python | <https://github.com/OSU-BMBL/RESEPT> |
|  |  | spaGCN | For integrating gene expression, spatial location, and histology to identify spatially variable genes (SVG), by graph convolutional network (GCN) | Python | <https://github.com/jianhuupenn/SpaGCN> |
|  |  | spaCell | For identifying cell types or predicting disease stages by integrating histopathological imaging analysis and transcriptomic analysis | Python | <https://github.com/BiomedicalMachineLearning/Spacell> |
| SVG identification | Hidden Markov Random Field | scGCO | For identifying genes demonstrating SVG using single-cell graph-cuts optimisation | Python | <https://github.com/WangPeng-Lab/scGCO> |
|  | Self-organising map NN | SOMDE | For finding gene spatial pattern based on Gaussian process accelerated by self-organising map | Python | <https://github.com/WhirlFirst/somde> |
|  | Gaussian process regression | spatialDE | An unsupervised, non-parametric, and automatic histology-based method | Python | <https://github.com/Teichlab/SpatialDE> |
|  | Generalised spatial model | SPARK | A non-parametric modelling enables scalable and robust detection of spatial expression patterns | Mainly R | <https://github.com/xzhoulab/SPARK> |
| Cellular communication | Random forest | MISTy | A multiview intercellular spatial modelling framework for profiling the intra- and inter-cellular relationship | R | <https://github.com/saezlab/mistyR> |
|  | Graph neural network (GNN) | NCEM | For learning cell communication from spatial graphs of cells toward understanding tissue phenotypes | Python | <https://github.com/theislab/ncem> |
|  | GCN | GCNG | For proposing extracellular interacting genes and assigning functional genes | Python | <https://github.com/xiaoyeye/GCNG> |
| Cell-type deconvolution | GCN | DSTG | For uncovering cell states and subpopulations based on spatial localisation | Mainly Python | <https://github.com/Su-informatics-lab/DSTG> |
|  | Modified adversarial discriminative domain adoption | CellDART | For cell-type inference by domain adaptation of single-cell and ST data | Python | <https://github.com/mexchy1000/CellDART> |
|  | Bayesian model | DestVI | Multi-resolution deconvolution of ST data by identifying continuous variation of the transcriptome within cells of the same type | Python | <https://github.com/romain-lopez/DestVI-reproducibility> |
|  | A collection of DL models | Tangram | For mapping single-cell transcriptomic data onto ST data | Python | <https://github.com/broadinstitute/Tangram> |
